# Improved Concrete Materials with Hydrogel-Based Internal Curing Agents

**DOI:** 10.3390/gels3040046

**Published:** 2017-11-25

**Authors:** Matthew J. Krafcik, Nicholas D. Macke, Kendra A. Erk

**Affiliations:** School of Materials Engineering, Purdue University, West Lafayette, IN 47907, USA; mkrafcik@purdue.edu (M.J.K.); nmacke@purdue.edu (N.D.M.)

**Keywords:** hydrogels, superabsorbent polymer (SAP), concrete, internal curing, gel-ion interactions, microstructure, poly(acrylic acid-acrylamide)

## Abstract

This research article will describe the design and use of polyelectrolyte hydrogel particles as internal curing agents in concrete and present new results on relevant hydrogel-ion interactions. When incorporated into concrete, hydrogel particles release their stored water to fuel the curing reaction, resulting in reduced volumetric shrinkage and cracking and thus increasing concrete service life. The hydrogel’s swelling performance and mechanical properties are strongly sensitive to multivalent cations that are naturally present in concrete mixtures, including calcium and aluminum. Model poly(acrylic acid(AA)-acrylamide(AM))-based hydrogel particles with different chemical compositions (AA:AM monomer ratio) were synthesized and immersed in sodium, calcium, and aluminum salt solutions. The presence of multivalent cations resulted in decreased swelling capacity and altered swelling kinetics to the point where some hydrogel compositions displayed rapid deswelling behavior and the formation of a mechanically stiff shell. Interestingly, when incorporated into mortar, hydrogel particles reduced mixture shrinkage while encouraging the formation of specific inorganic phases (calcium hydroxide and calcium silicate hydrate) within the void space previously occupied by the swollen particle.

## 1. Introduction

Portland cement is the most widely used building material in the world, and its production accounts for (5–10)% of the world’s total annual carbon dioxide emissions [1]. In the United States, 92 million metric tons of concrete were produced in 2015, contributing $10.6 billion to state revenues [2]. Thus, concrete is a massive worldwide industry and there is great potential for the incorporation of a wide array of material science solutions that increase the performance and sustainability of concrete materials. Such material advances would in turn reduce the need for repair and replacement of concrete pavement and infrastructure over time and result in further economic and environmental savings.

High performance concrete (HPC) is an advanced alternative to conventional concrete that possesses higher durability and strength due to its decreased porosity and also has lower carbon dioxide emissions [1,3]. HPC can easily achieve compressive strengths as high as 110 MPa, in contrast to the more common compressive strengths of (30–40) MPa [4]. The decreased porosity as well as disconnected pore network makes HPC quite resistant to corrosive fluid ingress and therefore extremely durable even in harsh environments [5]. Due to its lower permeability, HPC has in general good resistance to fire, although the smaller pore network may cause explosive spalling once sufficiently high temperatures are reached [6]. Such spalling can be mitigated through the addition of soft polymers, such as crushed rubber, as they impart a higher elastic modulus to the concrete [7].

HPC contains an excess of Portland cement relative to free water (low water-to-cement ratio), which results in a phenomenon known as self-desiccation. Water is consumed by the Portland cement to form calcium-silicate-hydrate (CSH), which is the inorganic binder from which the compressive strength of concrete is derived [8,9]. In HPC mixes, significant inward Laplace pressures will develop as the water is consumed and drained from the smallest pores within the hydrated cement network [10]. These pressures are high enough to cause a bulk volumetric collapse of the system referred to as autogenous shrinkage, which can result in early-age cracking and structural failure, especially if the concrete is restrained against forms, reinforcements, or other concrete [11].

To reduce self-desiccation and offset some of this early-age shrinkage, additional water can be supplied from external sources during the curing process (e.g., using hoses, sprinklers, or wet blankets). However, due to the dense microstructure and low permeability of HPC, it is difficult for external water to penetrate deep into the hardening cement matrix. Internal curing techniques offer a solution to this problem of HPC self-desiccation. One method of internal curing that has been explored more recently is the use of covalently crosslinked superabsorbent polymer (SAP) hydrogels [12,13,14,15,16,17]. These hydrogels are able to absorb and retain up to several hundred times their dry weight in fluid [18]. Hydrogels have been found to reduce autogenous shrinkage in HPC, and although some researchers have reported a decrease in early age strength after adding hydrogels, strength did recover to control levels with sufficient time [11,12,16,17,19]. The ability to control the mechanical response, swelling response, shape [20], and size of the hydrogels could also lead to hydrogels imparting multiple benefits to concrete. Hydrogels can be added dry to the cement mixture and relatively small amounts have been proven effective in reducing shrinkage of HPC (usually less than 2% by weight of cement), which makes the hydrogels more attractive to use than pre-soaked lightweight aggregates [21,22].

**Figure 1:**
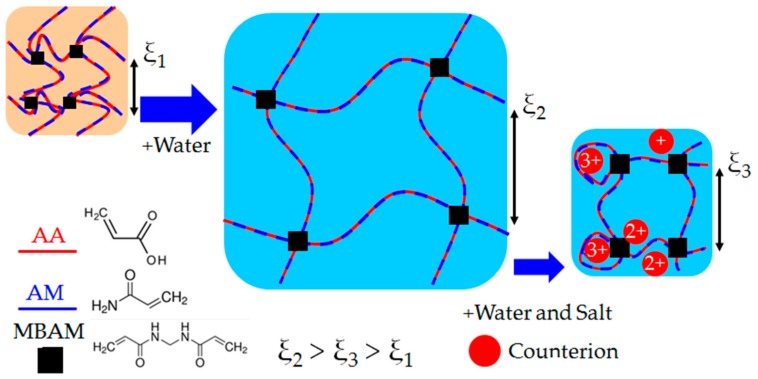
A schematic of a covalently crosslinked polyelectrolyte hydrogel network in a dry state (*ξ*_1_), swollen state (*ξ*_2_) after adding water, and deswollen state (*ξ*_3_) upon being exposed to a water and salt solution. The dry state is indicated with a lighter background colors. The approximate distance between crosslinks (mesh size) is indicated with *ξ*. The charged acrylic acid (AA) segments are indicated by is the dashed light red lines, while the uncharged acrylamide (AM) is indicated with dark blue lines. The covalent crosslinker (MBAM) is indicated with black squares, and counterions are indicated with red circles. The charges of each counterion are displayed within the circle. Monomer chemical structures (acrylic acid and acrylamide) are also provided. Not to scale.

A shortcoming of the research on polymer hydrogels for internal curing is that proprietary hydrogels with unknown chemical compositions are often used; frequently, even the monomers used to synthesize the hydrogels are not disclosed. The implicit assumption is made that the hydrogels are chemically inert within concrete materials. As shown in Figure 1, this assumption is not true for most hydrogels due to the interactions of the polyelectrolyte molecules that form the internal network of the hydrogel with mono- and multivalent ions that are naturally present in cementitious mixtures, including sodium, potassium, and calcium [23]. It has recently been shown [24] that a great deal more attention must be paid to the chemical structure as well as the method used for evaluating the swelling capacity of hydrogels when they are to be used as internal curing agents for concrete.

It is well known that when hydrogels are immersed in aqueous solutions, water and other nano-scale solutes (including counterions, surfactant, nanoparticles) will diffuse into the hydrogel’s internal polymer network as water is absorbed. Typically the network’s favorable interactions with water are due to the electronegativity of the polymer, allowing for certain chemical moieties to hydrogen bond with water molecules (e.g., amine groups) or electrostatically bond with polar molecules, including counterions in solution. As shown in Figure 1, the mesh size of the polymer network, *ξ*, effectively increases in the swollen state due to the flexible nature of the polymer chains.

For charged polymer networks, there is an additional driving force for diffusion of water and counterions into the gel [25]. For monovalent counterions in solution, the system resembles a Donnan membrane: the driving force for gel swelling can be identified with the swelling pressure, or net osmotic pressure, across a semipermeable membrane (here, the charged polymer network). Because the polymer network is charged and electroneutrality must be preserved, the concentration of free counterions within the hydrogel is higher than would be expected from ionic concentration alone (i.e., in the surrounding environment). Thus, the osmotic pressure of the solution inside the hydrogel will exceed that of the surrounding solution and the hydrogel will swell with additional fluid until the net osmotic pressure is zero.

In the highly alkaline environment of hydrating cement (with typical pH of 12–13, well above the pKa of acrylic acid, ≈4.25), the acrylic acid groups within the chemical structure of the hydrogel will deprotonate [26], allowing the resultant negatively charged moieties to complex with large amounts of water via ion-dipole interactions [27,28]. However, it is these same negative charges that can electrostatically interact with the various mono- and multivalent cations [29,30,31] (e.g., sodium, potassium, calcium) that are naturally found inside fresh cement mixtures as a result of the hydration reaction [23]. Figure 1 details the hydrogel network collapse (reduction in mesh size) that occurs as the polymer network coordinates with several types of counterions.

**Figure 2:**
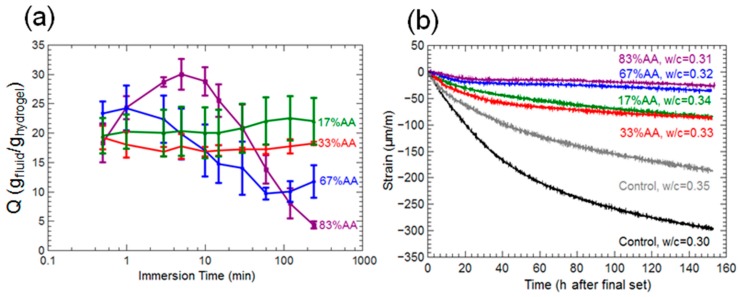
Hydrogel swelling ratios as a function of time in cement pore solution are shown in (**a**). Strain over time (autogenous shrinkage) results for cement mortars with and without hydrogels indicated by (**b**). Water-to-cement (w/c) ratios are provided for each mortar specimen in addition to hydrogel composition (% AA). Data adapted from Krafcik and Erk, 2016 [32].

In our previous study [32], it was clearly demonstrated that even if hydrogels were fully submerged in fluid, monovalent cations reduced swelling capacity and multivalent cations both significantly reduced maximum swelling capacity and changed swelling kinetics. The most important results from this study are summarized in Figure 2a. Interestingly, it was also shown that although the majority acrylic acid hydrogels displayed a fast deswelling response due to interactions with pore solution, these same hydrogels also provided the greatest reduction in autogenous shrinkage, indicated in Figure 2b.

Research within the polymer science community has found that trivalent cations, such as lithium and cesium, can bind permanently to charged polyelectrolyte hydrogels in contrast to mono- and divalent hydrogels, which can be removed from the hydrogel network [33]. Copper sulfate and silver nitrate solutions will form insoluble and stiff outer shells on polyacrylate hydrogels and can reconfigure the polymer network such that there is no longer a uniform distribution of polymer strands throughout the hydrogel. This results in a hydrogel sample with a thick outer “skin” of polymer and a hollow center [34]. Polymer chains can also self-assemble into ionically crosslinked gels when exposed to charged surfactants [35]. Such self-assembly of polyelectrolytes with oppositely charged ionic species is also reversible under the correct pH conditions [36]. Comparatively less research that examines the influence of aluminum on hydrogels has been done, although recent molecular dynamics simulations have shown that charged polymer chains will coil around trivalent cations much more tightly than around mono- and divalent cations [37]. Additionally, the lack of information in published literature about the chemical and physical structure of hydrogels used for cement internal curing can make it difficult if not impossible to discern the exact interactions that take place between the hydrogels and cement.

Within this work, two main investigations are described. First, we demonstrate that hydrogels are not chemically inert within cementitious systems and depending on hydrogel chemistry have significant impacts on cement paste microstructure. Second, we present experimental evidence that suggests the acrylic acid groups within hydrogels form strong associations with aluminum cations causing a reconfiguration of the hydrogel network as well as a loss of water from within the polymer structure. This work is intended to draw attention to the fact that charged polyelectrolyte hydrogels are not chemically inert in concrete applications. Due to the presence of multivalent cations within cement pore fluid, it is important that behaviors between hydrogels and cations be elucidated so that hydrogels are engineered to bring the most beneficial effects to a cementitious system. We synthesized hydrogels with differing ratios of acrylic acid (AA) and acrylamide (AM) using free radical solution polymerization. Four compositions were created (monomers are indicated in weight percent): 17 wt % AA–83 wt % AM, 33 wt % AA–67 wt % AM, 67 wt % AA–33 wt % AM, and 83 wt % AA–17 wt % AM. For brevity, each hydrogel will be referred to by the weight percent of AA only.

## 2. Results

### 2.1. Hydrogel Swelling in Salt Solutions

**Figure 3:**
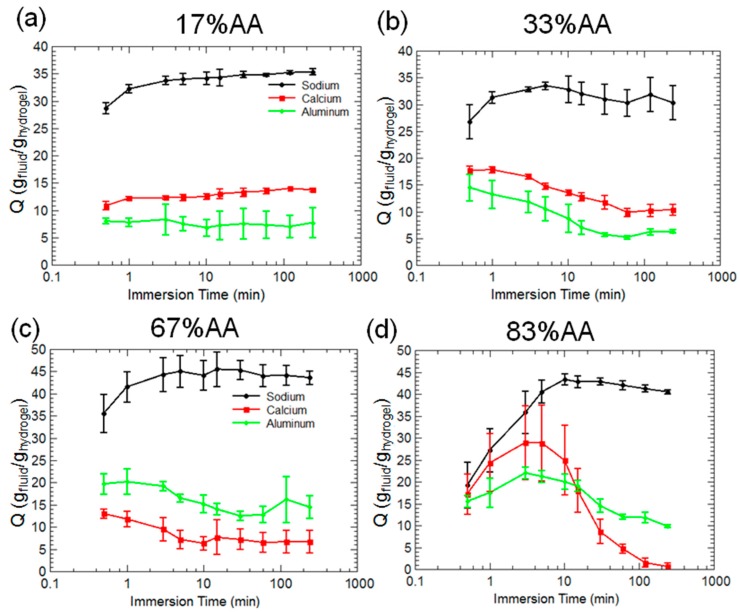
Swelling ratios over time for 17% AA (**a**), 33% AA (**b**), 67% AA (**c**), and 83% AA (**d**) hydrogels immersed in solutions of sodium chloride, calcium nitrate, and aluminum sulfate.

Figure 3 reports the swelling ratio, *Q*, of four types of hydrogels immersed in 0.025 M solutions of sodium chloride, calcium nitrate, and aluminum sulfate. These salts will dissociate in water leading to free sodium, calcium, and aluminum ions in solution. For the 17% AA acrylamide hydrogels in Figure 3a, equilibrium swelling ratios were reached within 5 min in the sodium solution, and equilibrium swelling is attained almost immediately in the calcium and aluminum solutions. Swelling capacity was markedly reduced when calcium and aluminum ions are present in solution. The 33% AA hydrogels in Figure 3b showed similar swelling in sodium and higher initial swelling in calcium and aluminum than 17% AA. However, deswelling was observed over time, and eventually the 33% AA hydrogels reached the same level as 17% AA in calcium and aluminum.

The 67% AA hydrogels in Figure 3c have similar swelling behavior to 33% AA, although the 67% AA did have higher swelling in sodium solution. An interesting property of the 67% AA hydrogels is the higher swelling capacity in aluminum solution, which is also shared for the 83% AA hydrogels at long times (>15 min). A markedly different swelling response over time was observed in Figure 3d for the 83% AA hydrogels, which displayed a peak swelling capacity in calcium and aluminum solutions at approximately 5 min, followed by a strong deswelling behavior. In the case of the calcium solution, all fluid was expelled from the hydrogels by 4 h.

### 2.2. Hydrogel Behavior in Aluminum Solutions

**Figure 4:**
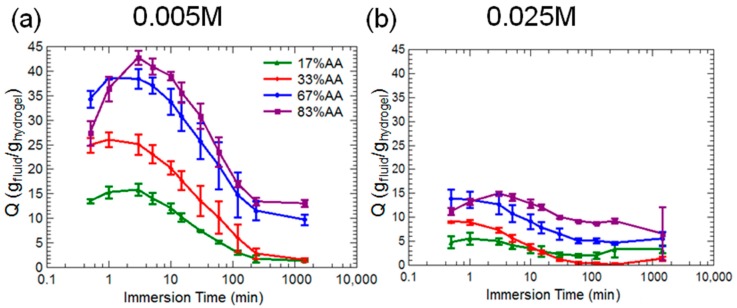
Swelling ratios over time for all hydrogel compositions in two different aluminum sulfate solution concentrations, (**a**) 0.005 M and (**b**) 0.025 M.

**Figure 5:**
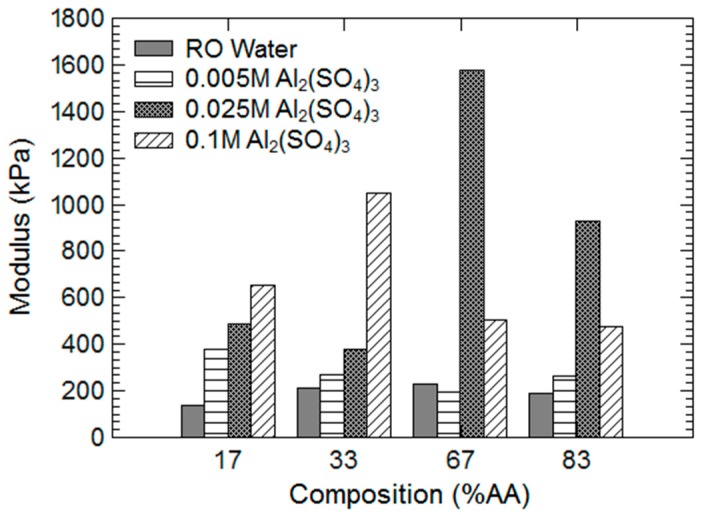
Elastic modulus of large hydrogel pieces that were exposed to several concentrations of aluminum sulfate solutions for 96 h. Modulus was calculated from the small-strain linear regime of the stress-strain curve obtained from compression testing.

**Figure 6:**
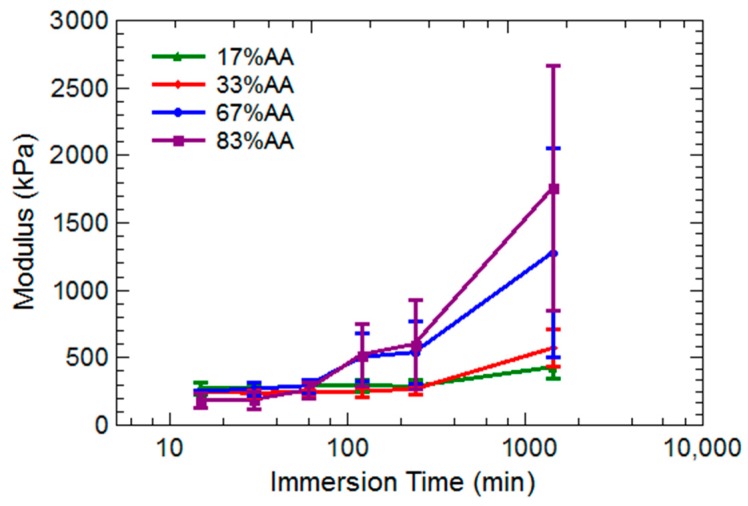
Elastic modulus as a function of time for all compositions of hydrogels immersed into 0.025 M aluminum sulfate solution. Higher uncertainties at later times are due to cracking, bulging, and other macroscopic deformations of the hydrogel specimen; modulus values after 24 h have been omitted from the graph.

To better understand the effects of aluminum on hydrogel mechanics and swelling response, several different molarity aluminum solutions were prepared and gravimetric measurements taken. Partial results are shown in Figure 4. Compressive strength measurements were performed on large hydrogel pieces that had been swollen in the aluminum solutions for 96 h to measure elastic modulus, and results are shown in Figure 5. Finally, elastic modulus as a function of time was measured for hydrogels swollen in 0.025 M aluminum solution, and these results are provided in Figure 6.

Figure 4 indicates that for all hydrogel compositions, maximum swelling ratios decreased as concentration of aluminum in solution increased. Swelling kinetics also changed. At 0.005 M concentration, all hydrogels displayed a peak swelling at short times (≈5 min) and then steady deswelling for the remainder of the test. At 0.025 M concentration, this behavior was still somewhat noticeable with the 83% AA hydrogels, but was not observed in the other three compositions. Virtually no swelling took place for all hydrogels in the 0.1 M solution, which is why these data have been omitted from the figure; all hydrogel compositions in the 0.1 M solution reached equilibrium swelling ratios at the beginning of the test and maintained a constant swelling ratio at approximately 4 ± 4 g_fluid_/g_hydrogel_.

Elastic modulus as a function of hydrogel composition and aluminum solution is shown in Figure 5. In reverse osmosis (RO) water, all hydrogel compositions were comparable in terms of elastic modulus, although the 17% AA hydrogel at approximately 150 kPa was slightly weaker than the others. In 0.005 M aluminum solution, only the 17% AA hydrogel displayed an increase in elastic modulus to approximately 400 kPa, with the other hydrogel compositions being about equal to RO water moduli (approximately 250 kPa) within uncertainties.

However, at 0.025 M aluminum concentration, all hydrogel compositions displayed a marked increase in elastic modulus, especially the majority acrylic acid hydrogel compositions (17 wt % AA, 33 wt % AA), with the 67% AA hydrogel having the highest elastic modulus of all at 1600 kPa. At 0.100 M aluminum concentration, the majority acrylamide hydrogel compositions (67 wt % AA, 83 wt % AA) continued to display an increase in elastic modulus, whereas the majority acrylic acid hydrogels show a substantial decrease in elastic modulus compared with immersion in 0.025 M solutions, and were about equal at 500 kPa.

Elastic modulus for all hydrogel compositions was measured over time immersed in a 0.025 M aluminum solution, and these results are presented in Figure 6. At times shorter than 100 min, elastic moduli of all hydrogels were essentially equivalent within uncertainties. At 120 min, the 67% and 83% AA hydrogels began to show an increase in elastic modulus. This was in contrast to the 17% and 33% AA hydrogels, which did not begin stiffening noticeably until 24 h during immersion.

The 67% and 83% AA hydrogels began to develop irregular cracks, curves, bulges, and bubbles that prevented the rheometer head from laying flush on the sample during compression testing measurements. No reliable data could be collected for these hydrogel compositions after 24 h. The 17% and 33% AA hydrogels did not display such morphologies, and continued to stiffen over time. The 33% AA hydrogel increased in elastic modulus from 250 to 1100 kPa whereas the 17% AA hydrogel increased from 250 to 600 kPa.

### 2.3. Influence of Hydrogel Chemistry on Cement Paste Microstructure

**Figure 7:**
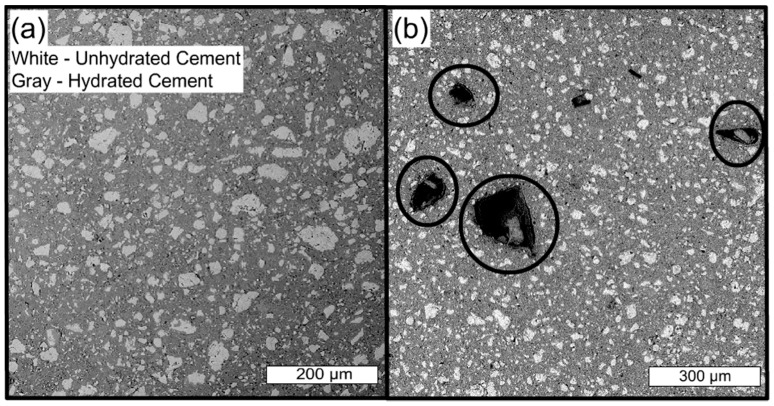
Comparison of control paste with no hydrogels (**a**) and 3-day cured paste containing 17% AA hydrogels (**b**). Each paste had w/c = 0.35. Hydrogel voids are indicated with black circles, unhydrated cement grains are white, hydrated product is the gray matrix throughout the image.

**Figure 8:**
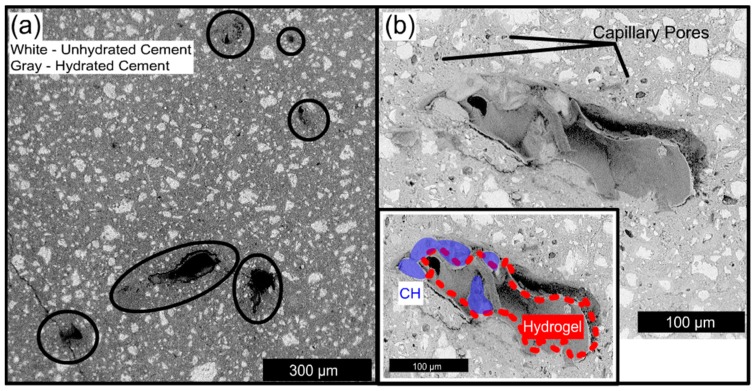
3-day cured pastes containing 67% AA hydrogels (**a**). Hydrogel voids are circled in black. Some voids have hydrated product in them. A 17% AA hydrogel void with capillary porosity surrounding it (**b**). Hydrogel remains are traced in red and calcium hydroxide crystals are indicated in blue (**b** insert).

Cement pastes with and without hydrogels were vacuum mixed and cured in limewater for 3 days. Pastes were sectioned and mounted in epoxy and then imaged with a scanning electron microscope in backscattered electron mode. This enabled quantification of void space as well as examination of phases with elemental detection via x-ray emission. Exemplary microstructures of control paste (no hydrogels, identical water-to-cement ratio) and a hydrogel paste are shown in Figure 7. Additional hydrogel paste and a close examination of a 17% AA hydrogel void along with phase analysis are provided in Figure 8. Even at low magnifications, hydrogel voids were clearly noticeable as shown in Figure 7b. Also noticeable is the presence of some hydrated product within several of the voids.

A number of striking features can be seen in Figure 8. First, the remains of hydrogel particles were visible in some of the voids. The hydrogel appeared as a grayish material that had separated from the wall of the void, and appeared to have uniformly changed in volume such that the shape of the hydrogel resembled the once fully occupied macrovoid. Similar microstructures of shell-like hydrogel morphology have recently been reported by Frazanian and Ghahremaninezhad [38] and macrovoids that retain the shape of the original swollen hydrogel were investigated with neutron tomography by Wyrzykowski, et al. [39]. Second, within (50–100) μm of some hydrogel voids, large capillary pores were visible, which strongly suggests that the hydrogels provided a locally higher water-to-cement ratio during curing. Similar results have been reported previously by Wehbe and Ghahremaninezhad [40]. Third, crystals of calcium hydroxide (CH) can be seen inside the hydrogel void. This likely indicates that the crystals were able to nucleate on the edge of the void and grow into the space previously occupied by swollen hydrogel, rather than grow into the surrounding hydrated cement. The composition was evaluated with energy dispersive x-ray analysis (EDX), and the CH formations are indicated in blue in the insert of Figure 8b. CH crystal growth is not observed in standard capillary porosity and also seems to be dependent on the chemical composition of the hydrogel.

**Figure 9:**
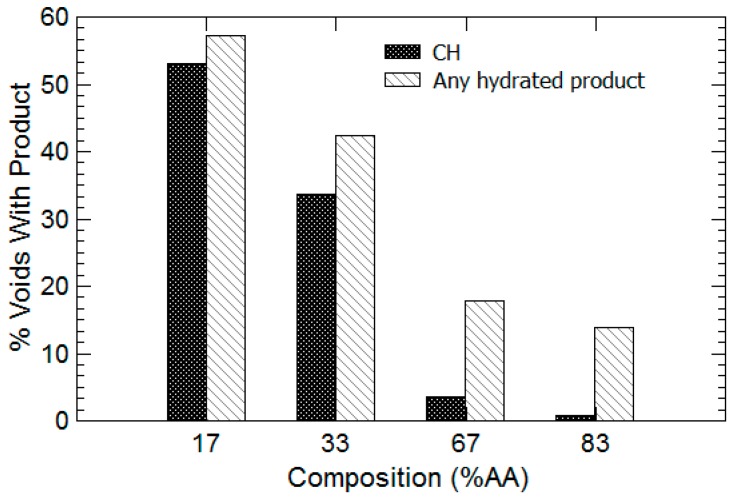
Percentage of total voids counted that contained either calcium hydroxide or some hydrated product (either calcium hydroxide and/or calcium-silicate-hydrate). As percent AA increases, the proportion of voids containing both calcium hydroxide and calcium-silicate-hydrate decreases dramatically.

To evaluate the dependence of CH formation on hydrogel chemistry, at least 100 voids were imaged from all areas of the sample surface at a magnification of 250× or greater. As shown in Figure 9, it is clear that as the amount of acrylamide increased in the hydrogel (and the percent AA decreased), the proportion of hydrogel voids containing either CH or calcium-silicate-hydrate (CSH) increased dramatically. Notably, the percentage of hydrogel voids containing CH increased from approximately 2% for 83% AA hydrogels to 34% for 33% AA hydrogels. CSH increases from 18% to 42% for the same two compositions, respectively. The amount of voids containing both CH and CSH continue to increase to approximately 52% and 58%, respectively, for the 17% AA hydrogel. Since EDX can only provide compositional information about the topmost portion of the evaluated area, there could be additional hydrated products, including CSH and perhaps ettringite, that may form in areas deeper within the hydrogel that cannot be seen by the microscope or investigated with EDX. Therefore, the data reported in Figure 9 are based entirely on the morphology of phases seen at the surface of the hydrogel void supplemented with available EDX data.

## 3. Discussion

### 3.1. Hydrogel Swelling Kinetics in Salt Solutions

The swelling kinetics displayed in Figure 3 are not entirely unexpected. Horkay and coworkers at the U.S. National Institutes of Health [33] conducted a number of investigations on the swelling behavior of poly(acrylic acid)-based hydrogels in water containing different ions, including sodium, potassium, and calcium. As concentration of ions was increased, the maximum swelling capacity of the hydrogel was reduced due in part to increased electrostatic screening of negative charges on the polymer backbone. Thus, the majority acrylamide hydrogels in Figure 3a,b were less sensitive to the effects of calcium and maintained higher swelling ratios than the majority acrylic acid hydrogels in Figure 3c,d. Swelling kinetics were dependent on the size of the hydrogels, with larger hydrogels taking longer times to reach equilibrium swelling ratios as well as displaying deswelling behavior. The 83% AA hydrogels were found to be slightly smaller in size than the 17% AA hydrogels, and so this could contribute to a more rapid swelling/deswelling in the case of the 83% AA hydrogels.

Hydrogels immersed in solutions containing divalent counterions displayed more rapid and discontinuous deswelling as a function of concentration than for monovalent solutions. It was also found that the polymer-solvent interaction (chi) parameter increased with increasing calcium concentration within the hydrogel, indicating that the free energy of hydrogels containing divalent ions has both ionic and mixing contributions [33]. This is in contrast to monovalent ions that only display ionic contributions to the total swelling pressure [29].

Interestingly, despite the strong interactions between polyanions and calcium, Horkay et al. [29] found no observed change in the shear modulus of the hydrogels, which indicated that divalent ions did not form stable ionic “bridges” that served to increase the effective crosslinking density of the gel. Rather, it was found that alkali earth metal ions (calcium, strontium) promoted weak aggregation of neighboring polymer chains. Although this collapse of polymer chains may not increase degree of crosslinking, it is certainly capable of expelling water from the hydrogel network, as indicated by Figure 3d. For majority acrylamide hydrogels, the spacing between deprotonated carboxylic acid groups may be far enough such that calcium ions cannot coordinate with multiple moieties. The opposite seems to be the case for the 83% AA hydrogels, as complete deswelling was observed at long times (4 h).

### 3.2. Hydrogel Swelling Behavior in Aluminum Sulfate Solutions

**Figure 10:**
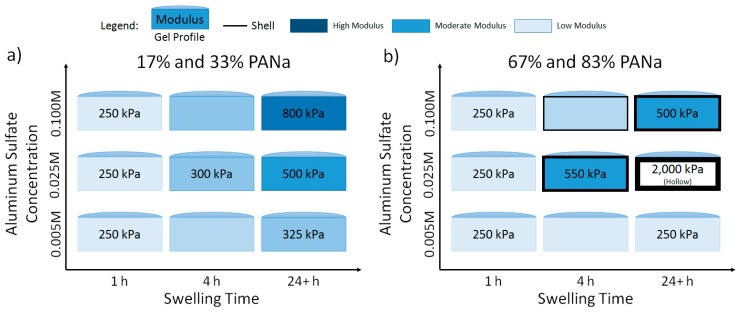
Morphology map that relates structure and mechanical properties of hydrogels as a function of aluminum sulfate concentration and immersion time. Majority acrylamide hydrogels are summarized in (**a**) and majority acrylic acid hydrogels are summarized in (**b**).

Figure 10 is a morphology map that connects the chemistry, morphology, and mechanical properties of hydrogels to immersion time and concentration of aluminum sulfate in solution. In general, the characteristics displayed by 17% and 33% AA hydrogels were similar as were the characteristics of 67% and 83% AA. Thus, two chemistries have been combined into each diagram. At 0.005 M aluminum concentration, the majority acrylamide hydrogels in Figure 10a displayed an increase in elastic modulus with time, and the effect was more pronounced with increasing aluminum concentrations. The majority acrylic acid shown in Figure 10b only displayed an increase in elastic modulus at and above the 0.025 M aluminum concentration and only after at least a four hours immersion time.

**Figure 11:**
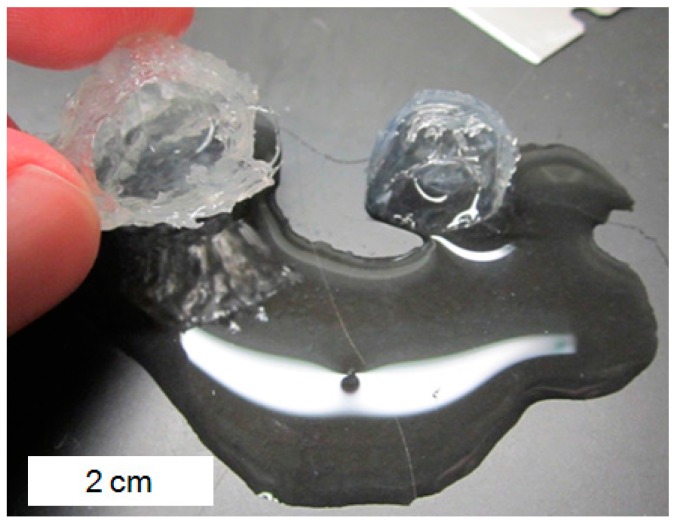
A photograph of a majority acrylic acid hydrogel several centimeters in size swollen in 0.025 M aluminum solution. The specimen was sliced in half with a razor blade, revealing a hollow, water-filled interior. This water was not bound to any part of the hydrogel and could be poured out of the hollow shell onto the table.

As indicated in Figure 10b, the majority acrylic acid hydrogels were observed to have the highest elastic modulus when immersed in the 0.025 M aluminum solution for long times. For majority acrylic acid hydrogels immersed for long times in 0.025 M and 0.1 M aluminum sulfate solutions, it was found that the macroscopic gel pieces used for compression testing had assumed an “eggshell-like” morphology, with a hollow, water-filled center (see Figure 11) and a mechanically stiff shell that was approximately (1–3) mm thick. The color of the hydrogels changed from completely transparent to a translucent white. Furthermore, the hydrogels could be sliced open with a razor blade and all of the water could be drained, as demonstrated in Figure 11. The mechanically stiff shell was found to be non-uniform in thickness and structural consistency, which led to aberrant elastic modulus data at longer immersion times (Figure 6). This was not the case for majority acrylamide hydrogels, which displayed more uniform strengthening throughout the entire gel.

The higher swelling ratios observed in Figure 3b for majority acrylic acid hydrogels in aluminum rather than calcium solution may be explained by the formation of this mechanically stiff outer shell. If the shell is dense enough to prevent the passage of solvent and forms before the entire polymer network has a chance to deswell and collapse, the hydrogels may be able to retain absorbed water better than when immersed in calcium, which does not form a dense layer to hinder the expulsion of solvent.

The rate of shell formation versus the rate of expulsion of water from the hydrogel network may explain why the majority acrylic acid hydrogels had higher overall swelling ratios than the majority acrylamde hydrogels as shown in Figure 3. Since aluminum is a trivalent ion, it can potentially coordinate with three negative groups on the polymer backbone, so it was anticipated that swelling ratio would be lowest in aluminum solutions for all hydrogel compositions. By immersing the hydrogels in various concentrations of aluminum sulfate, it was found that swelling kinetics were highly dependent on aluminum concentration. All hydrogel compositions displayed a short peak swelling followed by significant deswelling at 0.005 M (Figure 4a). Similar behavior is seen at 0.025 M (Figure 4b), although the changes in swelling capacities of all hydrogels were much lower, ranging of about 5 g_fluid_/g_hydrogel_ rather than (10–20) g_fluid_/g_hydrogel_ as observed in the 0.005 M concentration solutions. At 0.1 M, virtually no swelling was observed in any hydrogels, again suggesting that at high concentrations of aluminum, the cations are strongly associating with the outermost portions of the hydrogel and may be forming a dense layer through which the solvent cannot diffuse during the timescales of observation.

The presence of the mechanically stiff shell may suggest that the diffusion of aluminum ions into a hydrogel network is non-uniform and significantly different than the diffusion of sodium and calcium ions. Exposing the aluminum-soaked hydrogels to air or pure water for several days did not soften the hydrogels. It was not apparent that any absorbed aluminum had been removed from the shell, which suggests the ionic crosslinking of aluminum may be considerably stronger than that of calcium. This hypothesis is supported by results in Figure 5 and Figure 6, which clearly indicate that aluminum exposure caused an increase in elastic modulus of all hydrogel compositions, and the effect was most apparent for the 67% and 83% AA hydrogels.

Similar reductions in hydrogel swelling capacity along with the insoluble hollow-shell phenomenon were reported by Budtova and Navard [34] for polyelectrolyte hydrogels exposed to copper sulfate solutions. A later study [41] determined that while pure water could not dissolve the shell, a solution of hydrochloric acid could, suggesting replacement of copper ions with hydrogen atoms within the hydrogel network. Acid also caused further deswelling of the hydrogel once the copper atoms had been removed. Using confocal microscopy, Lapitsky and Kaler [35] have observed the self-assembly of solid and hollow gel beads following the exposure of flexible polyelectrolyte chains to charged surfactant molecules in solution. Depending on water, polymer, and surfactant concentration, a variety of phases were observed, including particles with a thin shell and sparse core, particles with stiff shells and ruptured interiors, and particles with stiff shells and the completely hollow interiors [35]. Such results are similar in principle to the variety of shell thicknesses and states of the hydrogel interiors that we report in Figure 10.

A 2014 study by Watanabe, et al. [42] used solvent exchange to create polymer capsules with internally porous structures ranging from tens to hundreds of microns, as well as to induce deswelling, formation of outer skins on the capsules, and phase separation. It was recently reported by Wehbe and Ghahremaninezhad [40] that the presence of shrinkage reducing admixtures (anionic surfactants) reduces the absorption capacity of hydrogels in cement pore solution, further suggesting that ionic complexing may be forming between charged surfactants and polyelectrolyte hydrogels within cementitious mixtures.

This behavior of hydrogel deswelling and hollow-shell formation was also seen for hydrogels exposed to surfactant by Göransson and Hansson [43], who found that the initial infusion of surfactant into the hydrogel was a diffusion-controlled process that did not immediately result in deswelling as expected. Rather, there seemed to be a metastable equilibrium between a two-phase system of internal gel and external hard shell. After this shell formed, diffusion of surfactant from the solution to the inside portion of the hydrogel was significantly hindered. A recent publication by Chremos and Douglas [37] indicated that trivalent cations caused charged polymer chains to “coil” around the counter-ions and become deformed. Interestingly, this coiling behavior also lead to an enrichment of counter-ions in the proximity of the coils and a reduction in the size of the counter-ion cloud that surrounds the polymer. This coiling may explain our observation of the hollow, water-filled shell shown in Figure 11 for majority acrylic acid hydrogels; the coiling of polymer chains around multiple aluminum cations may have been significant enough to concentrate the polymer network within the outer region of the hydrogel specimen while expelling water towards the interior of the hydrogel specimen, as the outer surface may have been too tightly bound with aluminum cations to allow the passage of water.

Huang, et al. [44], as well as Lawrence and Lapitsky [36], have utilized reversible ionic crosslinking between polyamines and multivalent anions to form stiff, adhesive, and self-healing gels. The amount of crosslinker required to form an effective gel was highly dependent on solution pH and ionic strength. Additionally, the ionic crosslinking was reversible based on solution pH. This result suggests that the shell on our hydrogels caused by exposure to aluminum may be removable if the hydrogels were immersed into a highly basic solution, in order that the aluminum ions could be replaced with hydrogen atoms. However, more testing is required to validate this hypothesis.

The molecular coiling, electrostatic shielding, and resulting collapse of polymer network chains in the presence of aluminum cations may explain why all composition of hydrogels in this study displayed similar swelling kinetics in aluminum sulfate solutions. The trivalent cations were able to bind with multiple moieties on the polyelectrolyte molecules and the resultant collapse in polymer network strands was enough to prevent the diffusion of water and other ions in solution. The multi-phase behavior of hydrogels in aluminum solutions as shown in Figure 10 is highly interesting and requires further study to obtain a more quantitative understanding of the relationships between aluminum concentration, hydrogel size, speed of shell formation, and shell thickness.

### 3.3. Influence of Hydrogel Chemistry on Cement Paste Microstructure

The results from the microstructure investigation on cement pastes containing hydrogels (Figure 7 and Figure 8) were both unexpected and encouraging. It was reported by Esteves, et al. [45] that poly(acrylic acid)-poly(acrylamide) copolymer hydrogels created a thermodynamically favorable environment for the precipitation of calcium hydroxide. Due to the higher number of negatively charged groups on majority acrylic acid hydrogels, it was initially expected that these hydrogels would absorb more calcium ions and thus have a higher percentage of hydrogel voids containing calcium hydroxide and CSH. Since the opposite result was observed here, it suggests that the presence of sufficient water may be apparently more important than the creation of calcium-rich areas within the hydrating cement slurry.

The 17% and 33% AA hydrogels did not display the significant deswelling due to ions that is characteristic of the 67% and 83% AA hydrogels, as shown in Figure 3c,d. Thus, the majority acrylamide hydrogels would be able to retain larger amounts of water for longer times. Combined with a slight absorption of calcium (as there are still some charged groups within the majority acrylamide hydrogels), the large water-filled hydrogel inclusions may create a favorable environment for the nucleation and growth of CH crystals. The majority acrylic acid hydrogels may desorb quite rapidly due to ionic interactions, and thus cannot provide sufficient water for the formation of hydrated product within the voids.

The fact that majority acrylamide hydrogels caused the formation of hydrated product within their voids is highly encouraging from an admixtures design perspective. If hydrogels have the ability to decrease porosity even partially, it suggests that the addition of hydrogels will not negatively impact the compressive strength of concrete. Furthermore, this evidence strongly suggests that hydrogel chemistry can be tuned to have multiple beneficial effects on concrete. If hydrogels can be engineered to refill void space with hydrated product, it could potentially offset any reduction in compressive strength that may be caused by the addition of the hydrogels.

## 4. Conclusions

In this study we have synthesized four different compositions of polyacrylate hydrogels and evaluated swelling kinetics in sodium, calcium, and aluminum solutions. The presence of sodium ions caused electrostatic shielding within the polymer network, leading to reduced swelling capacity. The calcium and aluminum were able to form ionic complexes with the polymer network leading to reduced swelling capacity, deswelling of the hydrogel over time, and in the case of trivalent aluminum ions, the formation of a mechanically stiff outer shell.

We examined hydrogel compressive strength as a function of hydrogel chemistry, immersion time, and aluminum concentration in solution. Whereas calcium ions will not form permanent bridges between charged moieties on the polymer backbone, aluminum ions appear to strongly associate with the polymer network, increase the elastic modulus (indicating an increase in the degree of crosslinking), and cannot be washed out with water. In majority acrylic acid hydrogels, the coiling and shielding effects of aluminum will create a mechanically stiff outer shell and hollow out the interior of the hydrogel, which indicates that aluminum ions may be damaging the polymer network, although more investigation is needed here.

We evaluated the influence of hydrogel chemistry on the formation of inorganic phases within hydrogel void space in hydrated cement paste. It was found that majority acrylamide hydrogels cause the formation of CH and CSH phases within hydrogel void space. Majority acrylic acid hydrogels had substantially less voids containing any type of hydrated product. This is a valuable result for the construction and building materials community, as it indicates that the organic chemistry of hydrogels used as internal curing agents can be tuned to have multiple effects on the local microstructure of cement and its resulting mechanical properties and performance. This could lead to the design of hydrogel-based materials that are multi-functional and will thus have a proportionally greater impact on the performance of concrete.

## 5. Materials and Methods

Hydrogels were created with acrylic acid (AA) and acrylamide (AM) monomers. The hydrogels were crosslinked at 2% by weight of monomer (AA + AM) with *N*-*N*′-methylenebisacrylamide (MBAM). The AA was partially neutralized with a solution of sodium hydroxide (NaOH). Two initiator solutions of sodium metabisulfate and sodium persulfate were used. All chemicals were used as-received from their respective companies. The NaOH was manufactured by Macron (Center Valley, PA, USA), the aluminum sulfate was manufactured by Fisher Scientific (Pittsburgh, PA, USA), the calcium nitrate was manufactured by Mallinckrodt Chemicals (St. Louis, MO, USA), and all other chemicals were obtained from Sigma-Aldrich (St. Louis, MO, USA). Reverse osmosis (RO) water with a total dissolved solid (TDS) content of approximately 15 ppm was used as the solvent for polymer preparation as well as the solvent for the swelling study. For the pastes, ordinary Portland Cement Type I (ASTM C150 [46]) supplied by Buzzi Unicem (Greencastle, IN, USA) was used. The Blaine fineness was 377 m^2^/kg, the loss on ignition was 1.34 wt %, and the oxide weight percents are as follows: 20.04 SiO_2_, 4.85 Al_2_O_3_, 3.35 Fe_2_O_3_, 63.5 CaO, 1.99 MgO. Tap water that had been tempered to room temperature was used for mixing pastes. Glenium 3030 NS full-range water reducer (WRA) manufactured by BASF (Ludwigshafen, Germany) was used at a concentration of 0.7% by weight of cement.

### 5.1. Hydrogel Synthesis

**Figure 12:**
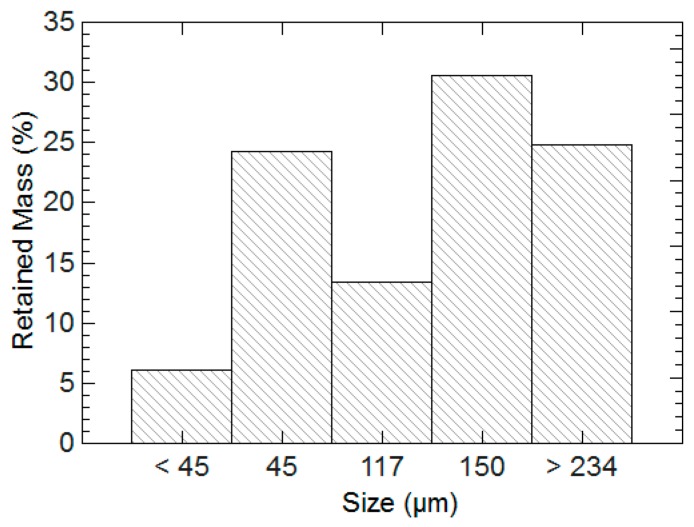
Amount of dry hydrogel mass that was retained on each sieve for the hydrogels synthesized for this study. Size indicates the maximum opening of the wire sieve [32].

The general recipe and exact proportions for polymer synthesis are listed in Table 1 [47]. Hydrogels were synthesized inside scintillation vials at room temperature. RO water, AA, and NaOH solution were added first. This addition of sodium hydroxide neutralized the carboxyl groups on the AA and formed sodium acrylate. The solution was allowed to thermally equilibrate for 10 min. AM and MBAM solution were then added to the vial. The solution was stirred for 10 min until all AM had dissolved. The two initiator solutions were added simultaneously, the vial was capped, shaken vigorously, and left stirring until a complete hydrogel had formed. Placing the vials in a thermal bath at 55 °C guaranteed that gelation occurred within 12 h of mixing the chemicals. 24 h after synthesis, the hydrogels were removed with a spatula from the vials and then washed for 24 h with RO water. After washing, the hydrogels were cut into small segments with a razor blade, dried in an oven for 12 h at 100 °C, and then ground to a fine powder with a pestle and mortar. An amount of dry hydrogels were placed on the top of wire sieves stacked from largest opening to smallest. The sieves were shaken, and the retained mass on each sieve was weighed. The resulting distribution was taken as an indicator of the hydrogel particle size, and this data is reported in Figure 12.

This synthesis method creates a single partially swollen gel within a scintillation vial. For hydrogels that were evaluated in compression testing, the scintillation vials were broken and several circular disks of hydrogel were cut from the main piece. The circular disks had a diameter of (2.5 ± 0.1) cm and thickness of (8 ± 2) mm. These disks were then sectioned into four curved triangular wedges with the same thickness and lengths of (1.2 ± 0.1) cm. These slices were either tested under compression immediately or were immersed into aluminum sulfate solutions. Each hydrogel piece was submerged in its own separate solution.

### 5.2. Swelling and Compression Tests

To obtain swelling ratios, hydrogels were evaluated in aluminum sulfate solutions of various molarities along with solutions of sodium chloride and calcium nitrate. Separate 0.005, 0.025 and 0.1 M solutions of anhydrous aluminum sulfate were prepared by adding the dry chemical to 200 mL RO water and stirring until it had dissolved completely. Each swelling test was repeated in triplicate to obtain an average and standard deviation.

For each solution prepared, an empty teabag was first placed into the solution for a few seconds until it became saturated with solution. The bag was then shaken to remove excess liquid and weighed to obtain the weight of the wet bag, *m_bag_*. The amount 0.2 g (*m_dry_*) of dry hydrogels was weighed and then placed inside the damp teabag. Equation (1) was used to calculate the swelling ratio, *Q*, at 30 s, 1, 3, 5, 10, 15, 30, 60, 120, 240 and 1440 min after immersion. Excess solution was removed before each weighing by holding the teabag against the side of the beaker until no more liquid was observed to be leaving the bag, and then the bag was weighed to obtain *m_wet_*.

(1) $$Q=\frac{m_{wet}-m_{dry}-m_{bag}}{m_{dry}}.$$

A TA Instruments AR-G2 rheometer was used for compression testing of hydrogels. The rheometer head was lowered onto the specimen and normal force was recorded by the machine. The test was displacement-controlled at 0.1 mm/s. Specimens were tested until failure or until the maximum compressive strength of the rheometer head (50 N) was reached. The linear portion of the stress-strain curve was identified and used to calculate elastic modulus. Partially swollen hydrogel pieces that were never exposed to aluminum were tested as controls.

Hydrogels exposed to aluminum sulfate solutions were tested under compression at the following time intervals: 5, 15, 30, 60, 120, 240 min, and 1, 2, 5 and 7 days. For each composition of hydrogel and aluminum solution (and for each time interval tested), at least three pieces of hydrogel exposed to identical conditions were tested in order to obtain mean and standard deviation of compressive strength and modulus results.

### 5.3. Paste Batching

Paste samples were created with a Renfert Twister Evolution vacuum mixer to ensure that any porosity would be due to capillary water and hydrogels. The mixture design is listed in Table 2. Dry cement that had been mixed with dry hydrogels (if applicable) was added to a cup. Water and WRA were added simultaneously, and the cup was placed onto the mixer. A vacuum was pulled, and the materials were mixed for 60 s. After the first mixing period, the cup was removed, sides scraped with a spoon, and left to sit for 45 s. Finally, the cup was reattached to the mixer and mixed for an additional 60 s under vacuum. The paste was then poured directly into a plastic mold, sealed, and placed in an environment chamber at (25 ± 1) °C and (50 ± 1)% relative humidity for 24 h. After 24 h, samples were removed from sealed conditions and placed into saturated limewater solutions for an additional 48 h. Samples were weighed before and after being removed from the chamber to ensure no mass loss had taken place during curing.

### 5.4. Backscattered Electron Microscopy

All microscopy specimens were cast into small cylinders and cured in sealed conditions for 72 h. The cylinders were then cut into small pieces with a saw, vacuum dried at (60 ± 5) °C for 72 h, and vacuum impregnated with a low-viscosity epoxy that was cured overnight at (60 ± 5) °C. The sample preparation method did cause macroscopic cracking as a result of drying shrinkage, and there may have been some decomposition of ettringite phases during the curing of the epoxy. These possible changes to microstructure were unavoidable since the presence of any free water and temperatures less than 60 °C prevents successful curing of the epoxy.

A fresh section of each epoxy sample was exposed with a diamond saw and the section was then polished with 3, 1 and 0.25 μm, polycrystalline diamond suspensions. All samples were carbon coated. Backscattered electron (BSE) images and elemental analysis from X-rays (EDX) were obtained with a NanoScience Instruments Phenom Desktop scanning electron microscope (SEM). For elemental analysis, point mapping with 15 kV accelerating voltage was used. From repeated measurements of cement phases with known compositions, it was determined that the quantitative analysis uncertainty is (3–5) weight percent. For pore size quantification, pictures were taken of the entire specimen surface and at least 100 voids from each sample were analyzed. Major and minor axes were measured and pores were evaluated with EDX if hydrated phases were seen.

## Figures and Tables

**Table 1:** Exact proportions of materials used for each hydrogel in this study. All items are in mL except for AM, which is in grams.

Hydrogel Type	AA	AM	Water	NaOH Soln.	Crosslinker Soln.	Init. Soln.
17 wt % AA	0.5	2.5	6.2	0.8	4.0	0.5 each
33 wt % AA	1	2	5.4	1.6	4.0	0.5 each
67 wt % AA	2	1	3.8	3.2	4.0	0.5 each
83 wt % AA	2.5	0.5	3	4.0	4.0	0.5 each

**Table 2:** Mixture proportions for pastes with and without hydrogels. Hydrogel dosage, water reducer (WRA) dosage, and total water contents are fixed across all samples. WRA is percent by weight of cement. *Q* are swelling ratios obtained in pore solution at 4 h after immersion.

Type	Cement (kg)	Water (kg)	w/c	Hydrogels (kg)	*Q*	WRA
Control	200	70	0.35	0.4	--	0.7
17 wt % AA	200	70	0.35	0.4	22	0.7
33 wt % AA	200	70	0.35	0.4	18.2	0.7
67 wt % AA	200	70	0.35	0.4	11.7	0.7
83 wt % AA	200	70	0.35	0.4	4.3	0.7
